# Early warning systems to predict severe complications during preterm and postpartum: A scoping review

**DOI:** 10.1111/jog.70079

**Published:** 2025-09-24

**Authors:** Eishin Nakamura, Katsuhiko Naruse, Ryuta Miyake, Tetsuya Hara, Marie Furuta, Hiroaki Tanaka, Shigetaka Matsunaga, Atsushi Sakurai, the Japan Resuscitation Council (JRC), Guideline Editorial Committee

**Affiliations:** ^1^ Center for Maternal, Fetal and Neonatal Medicine Saitama Medical Center, Saitama Medical University Kawagoe‐shi Saitama Japan; ^2^ Japan Resuscitation Council, Maternal Group Tokyo Japan; ^3^ Department of Obstetrics & Gynecology Dokkyo Medical University Shimotsuga‐gun Tochigi Japan; ^4^ Department of Obstetrics and Gynecology Nara Medical University Kashihara‐shi Nara Japan; ^5^ Department of Anesthesiology and Intensive Care Medicine Nagasaki University Graduate School of Biomedical Sciences Nagasaki Japan; ^6^ Department of Human Health Sciences Graduate School of Medicine, Kyoto University Kyoto Japan; ^7^ Department of Obstetrics and Gynecology Kumamoto General Hospital Yatsushiro Kumamoto Japan; ^8^ Division of Emergency and Critical Care Medicine, Department of Acute Medicine Nihon University School of Medicine Tokyo Japan

**Keywords:** early warning system, maternal death, postpartum hemorrhage, prediction model, scoping review

## Abstract

**Aim:**

Early warning systems (EWSs) are widely used in obstetric care to predict severe maternal complications, including postpartum hemorrhage (PPH) and sepsis. However, variations in the definitions of both EWS and target conditions hinder direct comparisons and meta‐analyses. This scoping review aimed to systematically identify diagnostic accuracy studies evaluating EWS for predicting severe maternal conditions and to explore trends in this field.

**Methods:**

This study followed the Preferred Reporting Items for Systematic reviews and Meta‐Analyses extension for Scoping Reviews guidelines. A systematic search of the MEDLINE/PubMed and CENTRAL databases was conducted up to July 12, 2024. Eligible studies included diagnostic accuracy studies assessing EWS in obstetric populations. Two reviewers independently screened studies, extracted data, and summarized study characteristics, including index tests and target conditions.

**Results:**

A total of 93 studies involving 697,558 patients were included. The most frequently evaluated index test was the shock index (SI; 38 studies, 41%), followed by the Modified Early Obstetric Warning Score (19 studies, 20.4%) and the Obstetric Early Warning Score (10 studies, 10.8%). PPH was the most common target condition (46 studies, 49.4%), followed by sepsis (30 studies, 32.3%) and maternal death (21 studies, 22.6%). The combination of SI and PPH was most frequently assessed (20 studies).

**Conclusions:**

Despite the widespread adoption of EWS in obstetric care, the marked heterogeneity in both index tests and outcome definitions highlights the need for standardized criteria and further diagnostic research.

## INTRODUCTION

Although maternal mortality has declined over the past few decades, it remains a significant public health issue, with 287,000 maternal deaths reported in 2020 according to the World Health Organization.^1^ Notably, 95% of these deaths occur in developing countries, highlighting the urgent need for effective preventive strategies. Among the leading causes of maternal mortality, postpartum hemorrhage (PPH) and sepsis account for a substantial proportion of cases; however, they are largely preventable with early detection and timely intervention.

Various scoring systems have been developed to assess the risk of severe maternal complications. The shock index (SI), the first such system, is a simple indicator used to detect maternal deterioration due to obstetric hemorrhage and has been widely adopted. Early warning systems (EWSs) that utilize vital signs and clinical symptoms are widely used in clinical practice.^2^ Some of these systems, such as the modified early warning score (MEWS) and obstetric early warning score (OEWS), can be implemented even in resource‐limited settings, making them valuable tools for the early detection of severe maternal conditions, including PPH and sepsis. Numerous studies have investigated the effectiveness of the EWS in predicting serious obstetric complications.

Despite their potential benefits, EWS face several challenges in both research and clinical applications. First, a wide variety of EWS are used in obstetric care; however, there is no universally accepted gold standard for risk assessment. Second, individual studies defined their own outcomes of interest, such as hemorrhage, sepsis, or massive transfusion, and reported the diagnostic accuracy accordingly. Third, the causes of maternal mortality are highly diverse and include PPH, sepsis, cardiovascular and cerebrovascular diseases, embolism, and pulmonary embolism, making it difficult for a single predictive model to comprehensively address all conditions. The Preferred Reporting Items for Systematic Reviews and Meta‐Analyses (PRISMA) guidelines for diagnostic test accuracy state that a meta‐analysis can only be performed if standardized and consistent definitions of the results are applied across all studies.^3^ Therefore, systematic reviews that integrate the diagnostic accuracy of EWS in obstetrics are difficult to conduct. Furthermore, there is a limited number of reports on the current situation.^4^

The aim of this study was to conduct a scoping review to systematically identify diagnostic accuracy studies on EWS for severe maternal complications and to highlight research gaps and future directions in this field.

## METHODS

### Protocol and registration

This study was designed and conducted in accordance with the PRISMA extension of the Scoping Reviews checklist.^5^ It was not pre‐registered, as scoping reviews are generally exempt from mandatory registration requirements. No written consent has been obtained from the patients as there is no patient identifiable data included.

### Eligibility criteria

Only articles published in academic journals were included in this review. Abstract‐only articles were included only if they clearly reported the sample size, index test(s), and outcome(s), enabling extraction of relevant diagnostic accuracy information. Studies were included if they had an English abstract, regardless of the language of the full text. Studies involving animals were excluded, and only those conducted on humans were considered. Additionally, studies that focused solely on healthy pregnant women were excluded.

The study types included randomized controlled trials and nonrandomized studies, including cohort and case–control studies. Review articles that used clinical studies as secondary data and systematic reviews were excluded.

The obstetric population was defined as pregnant women at ≥20 weeks of gestation or in the postpartum period, which was considered up to 6 weeks after delivery.

### Information sources

Articles published until July 12, 2024, were retrieved from MEDLINE/PubMed and CENTRAL. The search strategy was developed by public health experts and aimed at identifying diagnostic accuracy studies on EWSs in obstetric care. A literature search was conducted on July 12, 2024.

### Search

The full search strategy, including keywords and MeSH terms, is provided in Data S1, Supporting Information. The literature search covered studies from database inception through July 12, 2024.

### Selection of sources of evidence

The population, index tests, and target condition framework used in this study are presented in Table 1. This framework ensures a standardized approach for identifying relevant studies. For the index test, we included EWS based on established scoring systems and independently developed prediction models, including those that utilized machine learning. The target conditions were limited to those presented in Table 1; however, there have also been some reports of composite outcomes. For studies that reported composite outcomes, only those that counted the number of individual outcomes were included. Composite outcomes were included only if the study reported the number of each component outcome separately. That is, we excluded studies that reported composite outcomes without disclosing the breakdown of individual components (e.g., PPH, maternal ICU admission, or death).

**TABLE 1:** Population, index tests, and target condition (PIT) frameworks.

Population	Pregnant patients after 20 weeks gestation and postpartum patients.
Index tests	The following early warning systems are defined as index tests. Shock index (SI) Maternal early warning score (MEWS) Obstetrics early warning score (OEWS) Modified early obstetrics warning score/system (MEOWS) Maternal early warning criteria (MEWC) Maternal early warning trigger (MEWT) quick Sequential Organ Failure Assessment (qSOFA) Other independent prediction models, or machine learning
Target condition	Death Postpartum hemorrhage Pulmonary embolism Cerebrovascular disease Sepsis Massive blood transfusion Hypertensive disorder of pregnancy Eclampsia Cardiovascular disease Cerebrovascular disease Other severe maternal outcomes

The definitions of each index test are listed in Data S1.^6^, ^7^, ^8^, ^9^, ^10^, ^11^, ^12^, ^13^, ^14^ Each outcome was defined according to the criteria used in each study. For example, the definition of PPH varied between studies; however, because we did not plan to integrate diagnostic accuracy in this study, we extracted each outcome as defined and reported in the original study, without attempting to reclassify the definitions.

### Data charting process

Initially, two independent reviewers (Eishin Nakamura and Katsuhiko Naruse) screened titles and abstracts in a blinded manner (i.e., each reviewer was unaware of the other's decisions) using Covidence.^15^ Full‐text articles were likewise assessed independently. Any disagreements between the two reviewers were resolved through discussion, without the involvement of a third reviewer. The Excel‐based form was used solely for data charting (extraction) after study selection and was not used for screening. Study‐level information (e.g., sample size, index test[s], target condition[s], and outcome counts) was extracted using this predefined Excel form. Final inclusion decisions were made by consensus between the two reviewers, and no additional reviewer was involved in resolving discrepancies.

In addition, information on the country where each study was conducted was extracted, and each study was classified into World Bank 2024 income categories^16^ (high‐income, upper‐middle‐income, lower‐middle‐income, and low‐income).

### Data items

The extracted data included study sample size, type of index test used, details of the target conditions, and case counts. A complete list of the extracted data items is provided in Data S2.

### Critical appraisal of individual sources of evidence

Individual evidence supporting each indicator used to predict severe outcomes in pregnant and postpartum women is currently considered limited. Given the wide variety of available indicator tests, this scoping review may help reshape the perceptions regarding which tests are more appropriate for the target outcomes.

### Synthesis of results

We summarized the frequency of use of each index test for different obstetric outcomes. In addition, we categorized the index tests based on the target conditions to identify patterns in their applications.

## RESULTS

### Selection of sources of evidence

**FIGURE 1:**
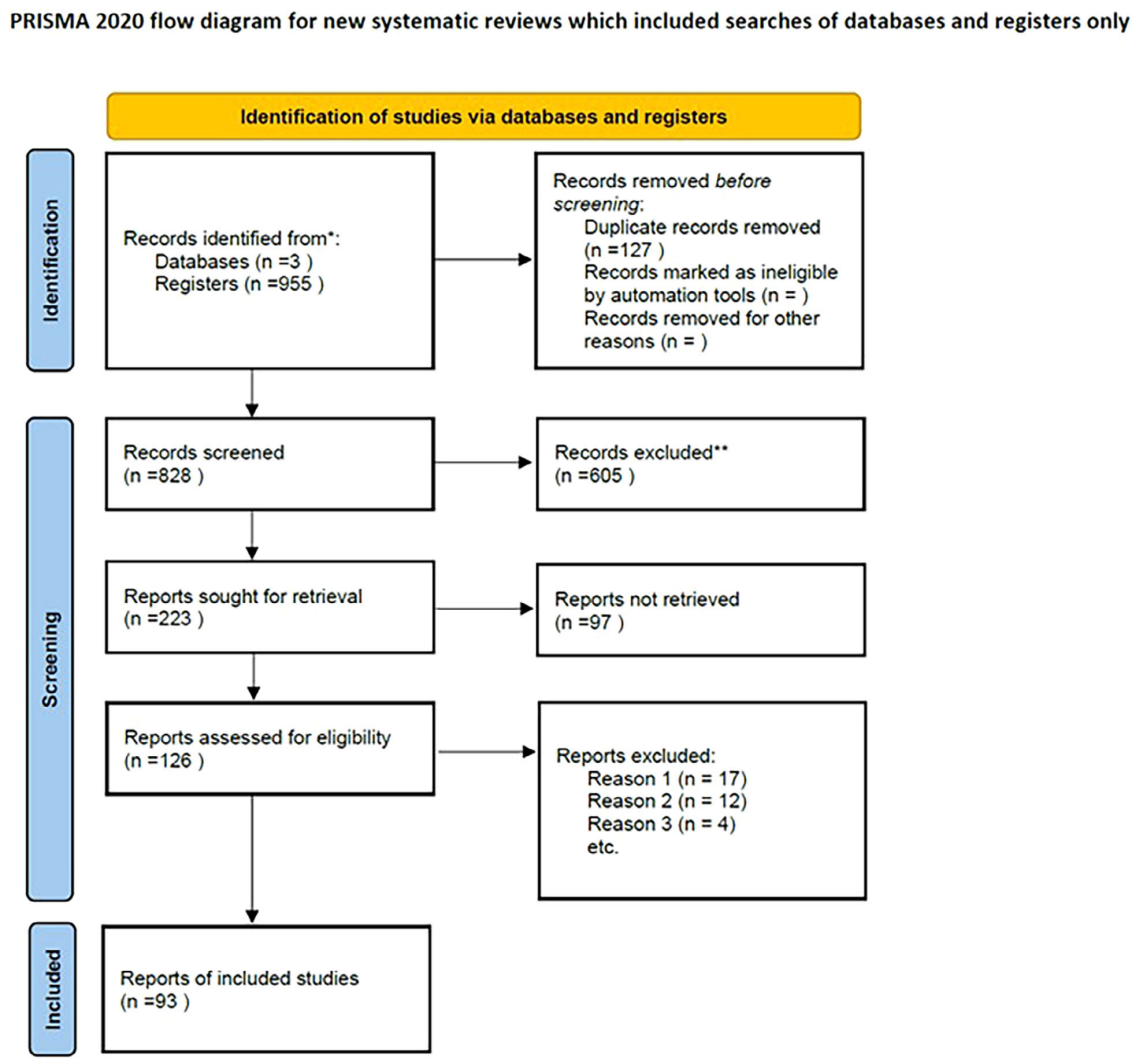
Preferred reporting items for systematic reviews and meta‐analyses (PRISMA) flowchart of the study.

Figure 1 shows the PRISMA flow diagram for this study. A total of 955 articles were identified through the literature search. After removing duplicates, 828 articles were retained for title and abstract screening. Of these, 223 articles underwent full‐text review, and 126 articles were initially deemed eligible.

Following further assessment, 33 articles were excluded for the following reasons: (i) 17 articles did not evaluate the predefined target conditions; (ii) 12 articles did not report a confirmable number of outcomes; and (iii) 4 articles did not utilize a diagnostic accuracy study design. A total of 93 articles met the inclusion criteria and were included in the final analysis.

### Characteristics of sources evidence

Table 2 provides an overview of the index tests examined in the final set of included studies. The 93 selected studies collectively involved 697,558 patients, who were assessed for the diagnostic accuracy of these tests. The SI was the most frequently used index test, appearing in 38 studies (41%). Modified early obstetric warning score (MEOWS) was the second most common, appearing in 19 studies (20.4%), followed by OEWS in 10 (10.8%). The remaining index tests were assessed in a more dispersed manner across studies.

**TABLE 2:** General characteristics of included studies with a focus on index tests.

Index test	Number of studies (*n* = 93)	Total patients (*n* = 697 558)
SI	38	82 349
MEOWS	19	29 635
OEWS	10	53 282
MEWS	9	54 552
MEWT	9	82 427
MEWC	6	10 532
qSOFA	6	18 703
Custom prediction models	6	322 846
YEARS	4	843
IVC diameter	3	486
Geneva	3	472
Modified SI	2	1495
MERC	2	2653
APACHE2	2	192
Age adjusted SI	1	883
omqSOFA	1	545
eCART	1	19 611
DNI	1	278
ONEWS	1	500
EMIP	1	69
SOS	1	15 055
Well's score	1	150

Abbreviations: APACHE2, acute physiologic assessment and chronic health evaluation version 2; DNI, delta neutrophil index; eCART, electronic cardiac arrest risk triage; EMIP, early maternal infection prompts; IVC, inferior vena cava diameter; MEOWS, modified early obstetrics warning score/system; MERC, maternal early recognition criteria; MEWC, maternal early warning criteria; MEWS, maternal early warning score; MEWT, maternal early warning trigger; OEWS, obstetrics early warning score; omqSOFA, obstetrically modified quick sequential organ failure assessment; qSOFA, quick Sequential Organ Failure Assessment; SI, shock index; SOS, sepsis in obstetric score.

We also summarized the geographical and socioeconomic settings of the included studies according to the World Bank 2024 income classification. Among the 93 studies, 66 (71.0%) were conducted in high‐income countries, 17 (18.3%) in upper‐middle‐income countries, 9 (9.7%) in lower‐middle‐income countries, and only 1 (1.1%) in a low‐income country. Detailed information on the country of origin and corresponding income classification for each study is provided in Data S2.

Table 3 summarizes the target conditions investigated in the included studies. PPH was the most frequently studied target condition (46 studies; 49.4%). It also accounted for the highest number of patients overall. Sepsis was examined in 30 studies (32.3%), whereas maternal death was assessed in 21 studies (22.6%). Among the 21 studies that included maternal death as a target outcome, 11 (52.4%) were conducted in high‐income countries, 3 (14.3%) in upper‐middle‐income countries, and 7 (33.3%) in lower‐middle‐income countries. No studies assessing death were conducted in low‐income countries.

**TABLE 3:** General characteristics of included studies with a focus on target condition.

Target condition	Number of studies (*n* = 93)	Total patients with target condition
Postpartum hemorrhage	46	10 600
Sepsis	30	3322
Massive blood transfusion	24	1182
Death	21	1467
Hypertensive disorder of pregnancy	18	2423
Pulmonary embolism	8	112
Eclampsia	4	54
Cardiovascular disease	3	201
Cerebrovascular disease	3	14

Table 4 summarizes the distribution of the index tests across different target conditions. The most frequently reported combination was the SI for PPH, which was reported in 20 studies. The MEOWS was the second most commonly assessed index test for PPH; however, fewer than five studies examined other index tests for this condition. Additionally, we observed frequent pairings, such as SI for massive blood transfusion (19 studies), MEOWS for sepsis (14 studies), and MEOWS for maternal death (7 studies), while other combinations were reported less frequently.

**TABLE 4:** Distribution of target conditions across index tests.

Index test	Death	Postpartum hemorrhage	Pulmonary embolism	Cardiovascular disease	Cerebrovascular disease	Sepsis	Massive blood transfusion	Hypertensive disorder of pregnancy	Eclampsia
SI	4	20	0	0	0	1	19	3	0
MEWS	3	3	0	0	0	6	2	1	1
OEWS	6	5	1	1	1	4	0	5	0
MEOWS	7	10	1	2	1	14	5	8	2
MEWC	2	2	0	0	0	2	1	3	0
MEWT	2	5	0	1	0	6	2	2	3
qSOFA	1	1	0	0	0	6	0	1	0
Custom prediction models	3	3	1	0	0	1	0	1	0
Modified SI	0	1	0	0	0	0	1	1	0
Age Adjusted SI	0	1	0	0	0	0	0	0	0
omqSOFA	0	0	0	0	0	1	0	0	0
eCART	1	0	0	0	0	1	0	0	0
IVC diameter	0	3	0	0	0	0	0	0	0
DNI	0	0	0	0	0	0	1	0	0
ONEWS	1	1	1	0	1	0	0	1	0
MERC	1	2	0	0	0	2	1	1	1
EMIP	0	0	0	0	0	1	0	0	0
SOS	0	0	0	0	0	1	0	0	0
APACHE2	1	1	1	1	1	2	1	0	0
Well's	0	0	1	0	0	0	0	0	0
Geneva	0	0	3	0	0	0	0	0	0
YEARS	0	0	4	0	0	0	0	0	0

Abbreviations: APACHE2, acute physiologic assessment and chronic health evaluation version 2; DNI, delta neutrophil index; eCART, electronic cardiac arrest risk triage; EMIP, early maternal infection prompts; IVC, inferior vena cava diameter; MEOWS, modified early obstetrics warning score/system; MERC, maternal early recognition criteria; MEWC, maternal early warning criteria; MEWS, maternal early warning score; MEWT, maternal early warning trigger; OEWS, obstetrics early warning score; omqSOFA, obstetrically modified quick sequential organ failure assessment; qSOFA, quick Sequential Organ Failure Assessment; SI, shock index; SOS, sepsis in obstetric score.

### Results of individual sources of evidence

Data S2 provides a detailed breakdown of each included study, listing the corresponding index test, target conditions, and total number of patients evaluated.

### Summary of evidence

In this scoping review, we systematically identified and analyzed diagnostic accuracy studies evaluating various index tests for severe obstetric complications between 2009 and 2024, ultimately including 93 studies. Our findings indicate that the number of diagnostic accuracy studies for each target condition is limited. Furthermore, substantial variability is observed in the target conditions assessed for each index test.

## DISCUSSION

This scoping review identified 93 diagnostic accuracy studies evaluating EWSs for predicting severe maternal complications, encompassing a total of 697,558 patients. The most frequently assessed index test was the SI, which might be due to its ease and simplicity of calculation, followed by the MEOWS and the OEWS. PPH is the most frequently studied target condition, and SI is the most commonly used predictive tool for PPH. However, substantial variability exists in the definitions of both index tests and target conditions, complicating direct comparisons across studies. These findings highlight the widespread use of EWS in obstetric care and underscore the need for greater standardization in diagnostic accuracy research.

Previous systematic reviews and meta‐analyses have examined the performance of individual EWS tools in obstetric populations. However, few studies have comprehensively mapped the landscape of diagnostic accuracy research in this field.^2^ Some studies have demonstrated the effectiveness of EWS in predicting severe maternal complications, whereas others have reported inconsistencies in their predictive value depending on the population and outcome definitions.^17^, ^18^ The lack of consensus regarding the gold standard, EWS, and variations in outcome measures remains major barriers to conducting meta‐analyses. Further research is needed to consolidate this evidence and establish standardized criteria for evaluating the EWS in obstetric settings.

A major strength of this study is its systematic approach for identifying and mapping the diagnostic accuracy of the EWS in obstetric care. By including a broad range of studies and categorizing index tests and target conditions, this review provides a comprehensive overview of the trends in the field. Notably, it highlights how different EWS tools have been applied to varying obstetric outcomes such as PPH, sepsis, and other critical conditions. This detailed mapping enables clinicians and researchers to identify the EWS that is predominantly used for predicting specific complications. Such insights can inform clinical decision‐making by helping practitioners select or interpret an EWS tool based on its most studied and potentially appropriate applications. For example, if a particular EWS has been primarily validated for predicting hemorrhage, clinicians may be more confident in using it to monitor patients at risk of that condition. Additionally, adherence to the Preferred Reporting Items for Systematic reviews and Meta‐Analyses extension for Scoping Reviews (PRISMA‐ScR) guidelines enhances the methodological rigor of this review.

However, this study had several limitations. First, heterogeneity in the definitions of index tests and target conditions limits direct comparability across studies. Second, the reliance on published studies with English abstracts may have introduced a selection bias, potentially omitting relevant research in other languages. Third, as this was a scoping review, no meta‐analysis was conducted to synthesize the pooled estimates of diagnostic accuracy. These limitations should be considered when interpreting the findings. Fourth, no formal risk‐of‐bias assessment was performed, and the methodological quality of included studies was heterogeneous. Therefore, the findings should be interpreted with caution.

Custom‐developed models and machine learning approaches were also included among the index tests identified in this review; however, these were highly heterogeneous in their development and application, and most lacked robust external validation. Therefore, their reported diagnostic performance should be interpreted with caution.

In addition, the geographical and socioeconomic distribution of the included studies showed a predominance of high‐income countries. This skew may limit the generalizability of our findings, particularly in low‐resource environments where maternal mortality is most pronounced. Notably, studies evaluating maternal death as an outcome were more frequently conducted in lower‐middle‐income countries compared with other outcomes. This distribution may reflect the higher burden of maternal mortality in resource‐limited settings, though the absolute number of such studies remains small.

## CONCLUSION

Despite the widespread adoption of EWS in obstetric care, substantial heterogeneity exists in the definitions and applications of index tests and target conditions. This variability poses challenges for evidence synthesis and the establishment of standardized clinical guidelines. Future research should focus on developing universally accepted definitions and evaluating the comparative performance of different EWS in diverse populations. Standardization efforts are critical for improving the accuracy and clinical utility of EWS in predicting severe maternal complications.

## AUTHOR CONTRIBUTIONS

**Eishin Nakamura:** Methodology; software; data curation; investigation; validation; formal analysis; project administration; visualization; writing – original draft; writing – review and editing. **Katsuhiko Naruse:** Data curation; methodology; software; investigation; supervision; resources; project administration; writing – review and editing. **Ryuta Miyake:** Data curation; investigation; validation; supervision; writing – review and editing. **Tetsuya Hara:** Data curation; supervision; investigation; validation; writing – review and editing. **Marie Furuta:** Writing – review and editing; resources; project administration; conceptualization; methodology; software; data curation; supervision. **Hiroaki Tanaka:** Conceptualization; supervision; writing – review and editing. **Shigetaka Matsunaga:** Conceptualization; supervision; writing – review and editing. **Atsushi Sakurai:** Conceptualization; supervision; project administration; resources; funding acquisition; writing – review and editing.

## CONFLICT OF INTEREST STATEMENT

Authors declare no conflict of interest for this article. Dr. Naruse Katsuhiko and Tanaka Hiroaki are an Editorial Board members of the JOGR Journal and co‐authors of this article. To minimize bias, they were excluded from all editorial decision‐making related to the acceptance of this article for publication.

## Supporting information


**Data S1:** Search strategy for this scoping review and definition of index test in this review.

**Data S2:** Complete detailed data of the study.

## Data Availability

Data Availability Statement All data analyzed in this scoping review are publicly available in the original studies cited in the manuscript. The authors did not generate or analyze any primary data. The data extraction sheet used for charting study characteristics is available from the corresponding author upon reasonable request.
