# Supplementary material for: Early warning systems to predict severe complications during preterm and postpartum: A scoping review
Source: J Obstet Gynaecol Res. 2025 Sep 24;51(9):e70079. doi: 10.1111/jog.70079 (PMC12582896; doi:10.1111/jog.70079)
Supplement: Supplementary file 1 — Data S1: Search strategy for this scoping review and definition of index test in this review. [file JOG-51-0-s001.docx]

1. **Search strategy for this scoping review**

Database: Ovid MEDLINE(R) <July Week 2 2024>

--------------------------------------------------------------------------------

1. exp Early Warning Score/

2. (shock adj2 (index or scor*)).mp.

3. (early adj2 warning adj2 (system* or scor* or criteri* or trigger*)).mp.

4. (MEOWS or MEWC or MEWT or SAAP).mp.

5. (rapid adj2 response adj2 (system* or recogni* or score* or monitor*)).mp.

6. geneva scor*.mp.

7. wells scor*.mp.

8. "sepsis-associated adverse outcomes in pregnancy score".mp.

9. 1 or 2 or 3 or 4 or 5 or 6 or 7 or 8

10. exp Maternal Mortality/

11. exp Pregnancy Complications/

12. exp Postpartum Hemorrhage/

13. exp Uterine Hemorrhage/

14. ((uterine or uterus or vaginal) adj2 (h?emorrhage* or bleed*)).mp.

15. exp Placenta Accreta/

16. exp Placenta Previa/

17. exp Embolism, Amniotic Fluid/

18. retained placenta.mp. or exp Placenta, Retained/

19. uterine atony.mp.

20. exp Disseminated Intravascular Coagulation/ or disseminated intravascular clotting.mp.

21. ((post partum or postpartum or postpartal or postnatal or post natal or puerperal or after childbirth or after birth or after giving birth or after delivery or after the delivery or following childbirth) adj3 (haemorrhag* or hemorrhag* or bleed* or bloodloss* or blood loss* or coagulopath*)).mp.

22. (abruptio placentae or metrorrhagia or placenta previa or anaphylactoid syndrome of pregnancy).mp.

23. ((uterus or intrauterine or "intra uterine" or uterine) adj3 (atony or atonic or inertia)).mp.

24. exp Puerperal Infection/

25. ((puerperal or postpartum or post-partum or post partum) adj2 (fever or infecti*)).mp.

26. ((amnioti* or amnion) adj3 (emboli* or embolu*)).mp.

27. 10 or 11 or 12 or 13 or 14 or 15 or 16 or 17 or 18 or 19 or 20 or 21 or 22 or 23 or 24 or 25 or 26

28. ((("Disseminated Intravascular" or disorder*) adj3 (coagula* or clot*)) or "DIC" or coagulopathy).mp.

29. exp Resuscitation/ or exp Cardiopulmonary Resuscitation/ or exp Resuscitation Orders/

30. exp Shock/

31. exp Sepsis/

32. ((cardiac or cardio* or cardiovascular or cardiopulmo* or cardio* or heart or thoracic) adj3 (arrest* or massage or compression)).mp.

33. (asystole or "pulseless electrical activity").mp.

34. exp Pulmonary Embolism/

35. exp Aortic Dissection/

36. ((aorta or aortic) adj1 (dissection or rupture)).mp.

37. exp Intracranial Hemorrhages/

38. ((brain or cerebral) adj3 (h?emorrhag* or breed* or blood*)).mp.

39. 28 or 29 or 30 or 31 or 32 or 33 or 34 or 35 or 36 or 37 or 38

40. exp Pregnancy/

41. exp Obstetrics/

42. exp Pregnant Women/

43. (prenan* or matern* or parturie* or obstetric* or birth* or post partum or postpartum or post-partum or intra partum or intrapartum or intra-partum or antenatal or postnatal or childbirth).mp.

44. 40 or 41 or 42 or 43

45. 39 and 44

46. 27 or 45

47. exp "Sensitivity and Specificity"/

48. exp "Predictive Value of Tests"/

49. ((positive or negative) adj "likelihood ratio").mp.

50. ((true or false) adj (positive or negative)).mp.

51. receiver operating characteristic.mp. or exp ROC Curve/

52. 47 or 48 or 49 or 50 or 51

53. 9 and (27 or 39)

54. 9 and (27 or (39 and 44))

55. 9 and (27 or 45 or 44)

56. limit 55 to human

****************************************************************************************

Search Name: CQ7CENTRAL

Date Run: 12/07/2024 13:22:24

Comment:

ID Search Hits

#1 MeSH descriptor: [Early Warning Score] explode all trees 11

#2 shock NEAR/2 (index or scor*) 289

#3 (early NEAR/2 warning NEAR/2 (system* or scor* or criteri* or trigger*)) 352

#4 MEOWS or MEWC or MEWT or SAAP 18

#5 (rapid NEAR/2 response NEAR/2 (system* or recogni* or score* or monitor*)) 45

#6 geneva NEXT scor* 11

#7 wells NEXT scor* 37

#8 "sepsis-associated adverse outcomes in pregnancy score" 0

#9 #1 or #2 or #3 or #4 or #5 or #6 or #7 or #8 731

#10 MeSH descriptor: [Maternal Mortality] explode all trees 199

#11 MeSH descriptor: [Pregnancy Complications] explode all trees 17886

#12 MeSH descriptor: [Pregnancy Complications] explode all trees 17886

#13 MeSH descriptor: [Postpartum Hemorrhage] explode all trees 992

#14 MeSH descriptor: [Uterine Hemorrhage] explode all trees 2446

#15 ((uterine or uterus or vaginal) NEAR/2 (h?emorrhage* or bleed*)) 4522

#16 MeSH descriptor: [Placenta Accreta] explode all trees 50

#17 MeSH descriptor: [Placenta Previa] explode all trees 82

#18 MeSH descriptor: [Embolism, Amniotic Fluid] explode all trees 3

#19 MeSH descriptor: [Placenta, Retained] explode all trees 88

#20 "retained placenta" 293

#21 "uterine atony" 301

#22 MeSH descriptor: [Disseminated Intravascular Coagulation] explode all trees 142

#23 "disseminated intravascular clotting" 243

#24 (("post partum" or postpartum or postpartal or postnatal or "post natal" or puerperal or "after childbirth" or after NEAR/3 birth or after NEAR/3 delivery or "following childbirth") NEAR/3 (haemorrhag* or hemorrhag* or bleed* or bloodloss* or "blood loss" or coagulopath*)) 3585

#25 ("abruptio placentae" or metrorrhagia or "placenta previa" or "anaphylactoid syndrome of pregnancy") 1097

#26 ((uterus or intrauterine or "intra uterine" or uterine) NEAR/3 (atony or atonic or inertia)) 362

#27 MeSH descriptor: [Puerperal Infection] explode all trees 163

#28 ((puerperal or postpartum or post-partum or "post partum") NEAR/2 (fever or infecti*)) 594

#29 ((amnioti* or amnion) NEAR/3 (emboli* or embolu*)) 45

#30 #10 or #11 or #12 or #13 or #14 or #15 or #16 or #17 or #18 or #19 or #20 or #21 or #22 or #23 or #24 or #25 or #26 or #27 or #28 or #29 25791

#31 ((("disseminated intravascular" or disorder*) NEAR/3 (coagula* or clot*)) or "DIC" or coagulopathy) 4147

#32 MeSH descriptor: [Resuscitation] explode all trees 7717

#33 MeSH descriptor: [Cardiopulmonary Resuscitation] explode all trees 1733

#34 MeSH descriptor: [Resuscitation Orders] explode all trees 57

#35 MeSH descriptor: [Shock] explode all trees 3482

#36 MeSH descriptor: [Sepsis] explode all trees 6523

#37 ((cardiac or cardio* or cardiovascular or cardiopulmo* or cardio* or heart or thoracic) NEAR/3 (arrest* or massage or compression)) 7695

#38 asystole or "pulseless electrical activity" 384

#39 MeSH descriptor: [Pulmonary Embolism] explode all trees 1534

#40 MeSH descriptor: [Aortic Dissection] explode all trees 216

#41 ((aorta or aortic) NEAR/1 (dissection or rupture)) 756

#42 MeSH descriptor: [Intracranial Hemorrhages] explode all trees 3178

#43 ((brain or cerebral) NEAR/3 (h?emorrhag* or breed* or blood*)) 17094

#44 #31 or #32 or #33 or #34 or #35 or #36 or #37 or #38 or #39 or #40 or #41 or #42 or #43 45334

#45 MeSH descriptor: [Pregnancy] explode all trees 34185

#46 MeSH descriptor: [Obstetrics] explode all trees 338

#47 MeSH descriptor: [Pregnant Women] explode all trees 1022

#48 prenan* or matern* or parturie* or obstetric* or birth* or "post partum" or postpartum or post-partum or "intra partum" or intrapartum or intra-partum or antenatal or postnatal or childbirth 109405

#49 #45 or #46 or #47 or #48 116771

#50 #44 and #49 3499

#51 #9 and (#30 or #50 or #49) 67

1. **Definition of index test in this review**
2. Shock index (SI)

The shock index (SI) is defined as the ratio of heart rate to systolic blood pressure, which measures the degree of shock.

1. Maternal early warning score (MEWS)

The Maternal Early Warning Score (MEWS) is used to monitor the vital signs of pregnant women and signs of deterioration in their clinical condition. The parameters generally included in the MEWS are heart rate, respiratory rate, blood pressure, and level of consciousness, and are tagged as normal values, yellow alerts, and red alerts.

1. Obstetrics early warning score (OEWS)

The Obstetric Early Warning Score (OEWS) is a clinical assessment tool developed to identify pregnant women at risk of clinical deterioration. It is calculated based on a combination of routinely measured vital signs including systolic and diastolic blood pressure, respiratory rate, heart rate, body temperature, and level of consciousness. In some versions, the required fraction of inspired oxygen (FiO₂) needed to maintain an oxygen saturation of 96% or higher is also included. By systematically evaluating these physiological parameters, the OEWS helps clinicians recognize early signs of critical illness and initiate timely interventions, even in resource-limited settings.

1. Modified early obstetric warning score/system (MEOWS)

The Modified Early Obstetric Warning Score (MEOWS) is a clinical tool designed to detect early signs of life-threatening illness in pregnant and postpartum women. It was calculated using five simple physiological parameters: mental status, pulse rate, systolic blood pressure, respiratory rate, and temperature. The underlying principle is that subtle changes across these variables, when assessed together, can signal deterioration earlier than when monitoring individual values alone. Among these, respiratory rate is considered the most sensitive indicator of a patient's physiological status, although it is often underrecorded in practice.

1. Maternal early warning criteria (MEWC)

The Maternal Early Warning Criteria (MEWC) were developed based on the Modified Early Obstetric Warning System (MEOWS) Red Triggers. The MEWC focuses on five key parameters: systolic and diastolic blood pressure, heart rate, respiratory rate, and oxygen saturation. Unlike earlier systems, the MEWC excludes temperature and pain because these variables are deemed less specific to severe maternal morbidity. Although fever typically coexists with other abnormalities and is unlikely to be overlooked in routine care, pain is considered a poor predictor of critical illness. Instead, MEWC includes oliguria as a clinical marker, which is particularly relevant in conditions such as preeclampsia with severe features. Urine output monitoring is recommended only for patients with clear indications such as suspected hemorrhage or major surgery. The threshold for bradycardia was raised from 40 to 50 beats per minute, and the need for increased oxygen supplementation, rather than low oxygen saturation alone, was used as a more specific indicator of respiratory compromise. Furthermore, the neurological criteria were broadened to include signs such as agitation, confusion, and persistent headaches in patients with hypertension.

1. Maternal early warning trigger (MEWT)

The Maternal Early Warning Trigger (MEWT) tool is a clinical system designed to aid in the early identification and management of maternal deterioration. This enables frontline healthcare providers, particularly nurses, to recognize the early signs of critical illness and promptly escape care through structured communication and response protocols. The MEWT focuses on four primary areas of maternal morbidity: hemorrhage, sepsis, hypertension-related complications, and cardiovascular dysfunction.

1. Quick Sequential Organ Failure Assessment (qSOFA)

The quick Sequential Organ Failure Assessment (qSOFA) is a simplified tool developed as part of the Sepsis-3 definitions to assist in the early identification of patients with suspected infection who are at an increased risk of poor clinical outcomes. It is intended for use outside intensive care settings and does not require laboratory tests, making it useful in emergency or low-resource environments. The qSOFA score is based on three clinical parameters: respiratory rate of 22 breaths per minute or higher, systolic blood pressure of 100 mmHg or lower, and any alteration in mental status. A score of two or more indicates a higher risk of mortality or prolonged ICU stay and warrants further clinical evaluation. Although originally designed for the general population, the qSOFA has been applied in obstetric settings, especially in the context of suspected maternal sepsis.
